# The crystal structure of bis­{3,5-di­fluoro-2-[4-(2,4,6-tri­methyl­phen­yl)pyridin-2-yl]phen­yl}(picolinato)iridium(III) and its 4-*tert*-butyl­pyridin-2-yl analogue

**DOI:** 10.1107/S2056989018012409

**Published:** 2018-09-21

**Authors:** Robert D. Sanner, Victor G. Young

**Affiliations:** a Lawrence Livermore National Laboratory, Livermore, CA 94550, USA; b Department of Chemistry, University of Minnesota, Minneapolis, MN 55455, USA

**Keywords:** crystal structure, organic light-emitting diode, organometallic light emitter, luminescent iridium complex, polymorph, cyclo­metallated iridium complex

## Abstract

The crystal structures of bis­{3,5-di­fluoro-2-[4-(2,4,6-tri­methyl­phen­yl)pyridin-2-yl]phenyl-κ^2^ *N*,*C* ^1^}(picolinato-κ^2^ *N*,*O*)iridium(III), [Ir(C_20_H_16_F_2_N)_2_(C_6_H_4_NO_2_)], **1**, and bis­[2-(4-*tert*-butyl­pyridin-2-yl)-3,5-di­fluoro­phenyl-κ^2^ *N*,*C* ^1^](picolinato-κ^2^ *N*,*O*)iridium(III), [Ir(C_15_H_14_F_2_N)_2_(C_6_H_4_NO_2_)], **2**, are presented herein. These phospho­rescent cyclo­metallated iridium(III) compounds have been structurally investigated in order to better understand the nature of their blue-shifted emssions while maintaining high quantum yields. Compound **1** exhibits substantial twisting of the mesitylene rings out of the plane of the attached pyridine ring, with dihedral angles of 67.0 (1) and 78.7 (1)° between the best mean planes. For both compounds, the contribution of disordered solvent mol­ecule(s) was removed using the SQUEEZE [Spek (2015 ▸). *Acta Cryst.* C**71**, 9–18] routine in *PLATON* [Spek (2015 ▸). *Acta Cryst.* C**71**, 9–18]. These solvent mol­ecules are not considered in the given chemical formula and other crystal data.

## Abstract 2

The crystal structures of two blue-emitting iridium(III) cyclo­metallates were determined and related to the photophysical properties of the complexes.

## Chemical context   

Phospho­rescent cyclo­metallated iridium(III) compounds have been investigated for a variety of applications, including solar cells (Kim *et al.*, 2016 ▸), sensors (Marín-Suárez *et al.*, 2012 ▸), bioimaging (Zhang *et al.*, 2015 ▸), and scintillators (Bertrand *et al.*, 2015 ▸). However, their most widespread use stems from their electro­phospho­rescence and research in this area has focused on their use in organic light-emitting diodes (OLED) (Choy *et al.*, 2014 ▸; Chi & Chou, 2010 ▸; Fu *et al.*, 2011 ▸). The most widely studied blue-emitting iridium complex for this purpose is bis­[2-(4,6-di­fluoro­phen­yl)pyridinato]iridium(III) picolinate, or FIrpic (Baranoff & Curchod, 2015 ▸). As part of our synthesis program for plastic scintillators (Rupert *et al.*, 2012 ▸; Cherepy *et al.*, 2015 ▸), we have prepared and structurally characterized several blue-emitting iridium complexes (Sanner *et al.*, 2016 ▸). A recent study by another group has examined the attachment of alkyl or aryl groups to the pyridine ring of the (di­fluoro­phen­yl)pyridinate ligand in FIrpic (Kozhevnikov *et al.*, 2013 ▸), resulting in enhanced quantum efficiency, while maintaining the sky-blue color of the parent complex. We have prepared and structurally characterized two of those complexes to examine the structural basis for their superior behavior. We report herein the crystal structures of bis­{3,5-di­fluoro-2-[4-(2,4,6-tri­methyl­phen­yl)pyridin-2-yl]phenyl-κ^2^ *N*,*C* ^1^}(picolinato-κ^2^ *N*,*O*)iridium(III), **1**, and bis­[2-(4-*tert*-butyl­pyridin-2-yl)-3,5-di­fluoro­phenyl-κ^2^ *N*,*C* ^1^](picolinato-κ^2^ *N*,*O*)iridium(III), **2**. The mol­ecular structure of **2** has been reported previously (Laskar *et al.*, 2006 ▸), however, the structure reported below is a new polymorph.
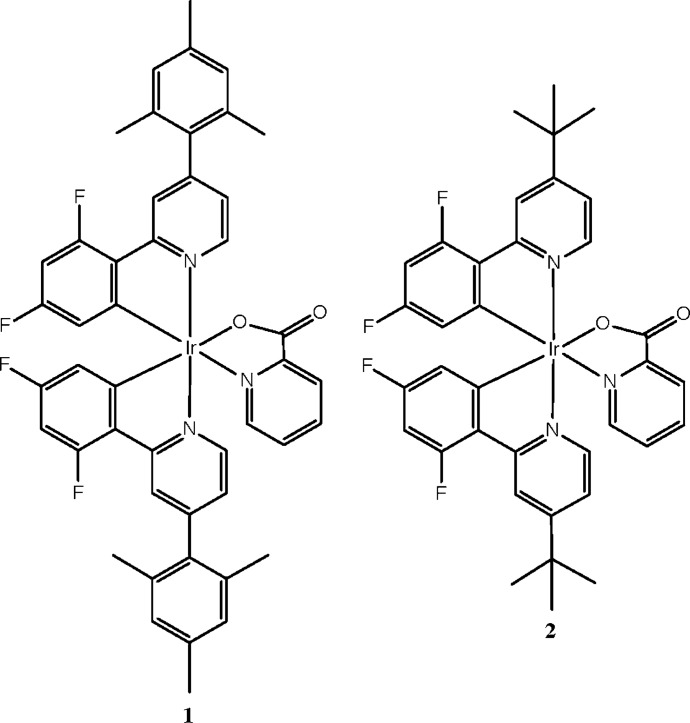


## Structural commentary   

**Figure 1:**
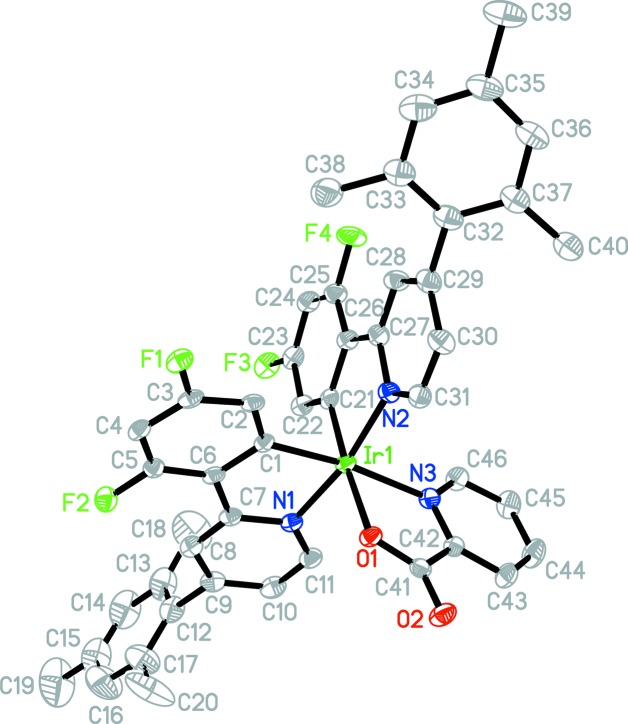
The mol­ecular structure of **1**, with the atom labeling and displacement ellipsoids drawn at the 50% probability level. H atoms have been removed for clarity.

The mol­ecular structure of complexes **1** and **2** have been confirmed by X-ray crystallography and displacement ellipsoid diagrams are shown in Figs. 1 ▸ and 2 ▸. Both complexes exhibit the same distorted octa­hedral geometry with two bidentate phenyl­pyridine ligands (coordinated through the pyridine N atom and a phenyl C atom) and one bidentate pyridine-2-carboxyl­ate ligand, also known as 2-picolinate (coordinated through the pyridine N atom and a carboxyl­ate O atom). The iridium-bound N atoms of the phenyl­pyridine ligands are *trans* to each other, while the phenyl C atoms bound to iridium are *cis*. The two ligated atoms in the picolinate ligand are then *trans* to the phenyl C atoms of the phenyl­pyridine ligand. The *trans* effect of the phenyl groups is most clearly seen when comparing Ir—N bond lengths. Thus, the Ir—N(picolinate) bond is on average 0.1 Å longer than the Ir—N(phenyl­pyridine) bond due to its *trans* disposition to the phenyl group. Although there is no such intra­molecular comparison available for the picolinate Ir—O bond (which is also *trans* to a phenyl group), we note that the Ir—O bond length in both these mol­ecules is 2.16 Å. This compares with the shorter value of 2.09 Å reported in the Cambridge Structural Database (CSD; Version 5.39, May 2018, with three updates; Groom *et al.*, 2016 ▸) for an Ir—O bond, again illustrating the *trans* effect of the phenyl group in these mol­ecules. The C—Ir—N ‘bite’ angle for the phenyl­pyridine ligand averages 80.8 (4)° for these complexes, while the N—Ir—O angle for the picolinate ligand is somewhat smaller at 76.7 (2)°. The phenyl and pyridine rings in each phenyl­pyridine ligand are slightly twisted with respect to each other across the C—C bond linking the two rings. The dihedral angle between the best planes for the two rings is in the range 6–10° in these mol­ecules. A feature of special inter­est in **1** is the dihedral angle between the plane of the pyridine ring and that of the attached mesityl group, *e.g.* between the N1/C7–C11 ring and the C12–C17 ring. These values are 67.0 (1) and 78.7 (1)° for the two mesit­yl–phenyl­pyridine ligands in **1**. The presence of two *ortho*-methyl groups on the mesitylene (*e.g.* C18 and C20) presumably causes this large twist of the mesityl ring out of the plane of the attached pyridine ring. This possibility has been proposed (Kozhevnikov *et al.*, 2013 ▸) as an explanation for the blue emission of **1** since it minimizes the π-conjugation between the mesityl and pyridine rings which would otherwise lead to red-shifted emission. Our results confirm the structural basis for this proposal.

## Supra­molecular features   

Neither complex forms any significant supra­molecular inter­actions with neighboring mol­ecules.

## Database survey   

The mol­ecular structure of **2** has been reported previously (Laskar *et al.*, 2006 ▸; CSD refcode CEHGOM); however, the structure reported below is a new polymorph as a solvate. A search of the CSD (Groom *et al.*, 2016 ▸) provided no additional crystal structures related to **1** or **2**.

## Synthesis and crystallization   

The title compounds were prepared as described previously (Kozhevnikov *et al.*, 2013 ▸). Diffraction-quality crystals of **1** were obtained by slow evaporation using methanol as solvent, while **2** utilized a 1:2 (*v*/*v*) methyl ethyl ketone–hexane mixture as solvent.

## Refinement   

Crystal data, data collection and structure refinement details are summarized in Table 1 ▸. All H atoms were placed in calculated positions and refined as riding atoms; for aryl H atoms, C—H = 0.95 Å and *U*_iso_(H) = 1.2*U*_eq_(C), and for methyl H atoms, C—H = 0.98 Å and *U*_iso_(H) = 1.5*U*_eq_(C). Both specimens used for this study contained badly disordered solvent of crystallization. Specimens for compound **1** tended to lose solvent during mounting. The initial structure solution appeared to be either a methanol tris­olvate or a methanol/water disolvate, where all three mol­ecules were concatenated through hydrogen bonds to the O2 hydrogen-bond acceptor. Only one methanol solvent mol­ecule was clearly indicated, but it had considerable translational displacements toward a putative compositionally disordered methanol/water site. The last member of this chain was likely a methanol, but it was pathologically disordered. The SQUEEZE routine (Spek, 2015 ▸) from *PLATON* (Spek, 2009 ▸) was applied to this disordered solvent region since in least-squares no reasonable disorder model could be achieved. Void spaces centered at (0, 0, 0) and (0, 0.5, 0.5) totaling 727 Å^3^ were found to contain an electron count of 177. This electron count would correspond to approximately ten methanol mol­ecules per unit cell. The specimen for compound **2** did not appear to lose solvent during mounting. The initial structure solution found the expected compound and a region near an inversion center composed of unknown solvent. The peaks in the difference Fourier map did not provide any reasonable solvent mol­ecule (or mol­ecules) after numerous attempts. The SQUEEZE routine from *PLATON* was applied to this disordered solvent region. Void spaces centered at (0, 0, 0.5) and (0, 0.5, 0) totaling 954.7 Å^3^ were found to contain an electron count of 203. This electron count would correspond to approximately four methyl ethyl ketone or hexane mol­ecules per unit cell.

## Supplementary Material

Crystal structure: contains datablock(s) 1, 2. DOI: 10.1107/S2056989018012409/lh5880sup1.cif

Structure factors: contains datablock(s) 1. DOI: 10.1107/S2056989018012409/lh58801sup2.hkl

Structure factors: contains datablock(s) 2. DOI: 10.1107/S2056989018012409/lh58802sup3.hkl

CCDC references: 1865394, 1865393

Additional supporting information:  crystallographic information; 3D view; checkCIF report

## Figures and Tables

**Figure 2:**
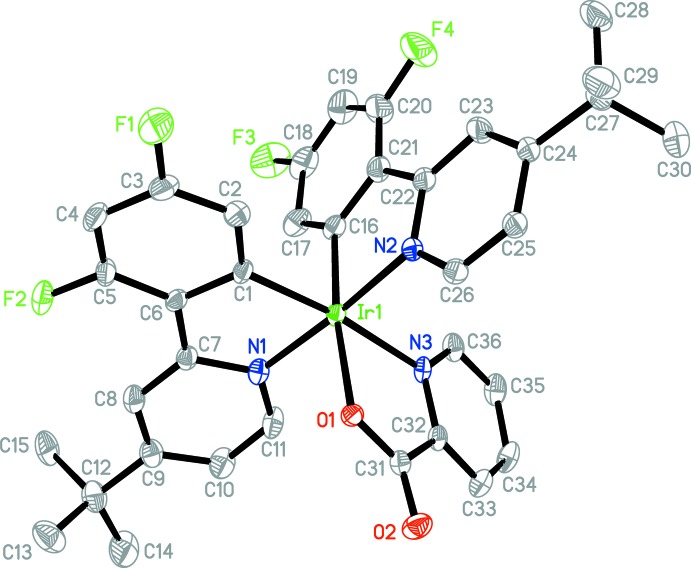
The mol­ecular structure of **2**, with the atom labeling and displacement ellipsoids drawn at the 50% probability level. H atoms have been removed for clarity.

**Table 1:** Experimental details

	**1**	**2**
Crystal data		
Chemical formula	[Ir(C_20_H_16_F_2_N)_2_(C_6_H_4_NO_2_)]	[Ir(C_15_H_14_F_2_N)_2_(C_6_H_4_NO_2_)]
*M*_r_	930.98	806.84
Crystal system, space group	Monoclinic, *P*2_1_/*c*	Monoclinic, *P*2_1_/*c*
Temperature (K)	100	100
*a*, *b*, *c* (Å)	15.1582 (12), 12.3530 (9), 24.2353 (17)	14.7519 (7), 14.4121 (7), 18.5425 (8)
β (°)	106.374 (3)	104.7761 (19)
*V* (Å^3^)	4354.0 (6)	3811.9 (3)
*Z*	4	4
Radiation type	Mo *K*α	Mo *K*α
μ (mm^−1^)	3.12	3.55
Crystal size (mm)	0.12 × 0.12 × 0.12	0.15 × 0.13 × 0.04

Data collection		
Diffractometer	Area Bruker PHOTON-II CPAD	Area Bruker PHOTON-II CPAD
Absorption correction	Multi-scan (*SADABS*; Bruker, 2014 ▸)	Multi-scan (*SADABS*; Bruker, 2014 ▸)
*T*_min_, *T*_max_	0.576, 0.746	0.370, 0.433
No. of measured, independent and observed [*I* > 2σ(*I*)] reflections	74054, 13464, 11535	81284, 11662, 9342
*R*_int_	0.042	0.049
(sin θ/λ)_max_ (Å^−1^)	0.718	0.715

Refinement		
*R*[*F* ^2^ > 2σ(*F* ^2^)], *wR*(*F* ^2^), *S*	0.030, 0.063, 1.10	0.025, 0.057, 1.02
No. of reflections	13464	11662
No. of parameters	512	421
H-atom treatment	H-atom parameters constrained	H-atom parameters constrained
Δρ_max_, Δρ_min_ (e Å^−3^)	2.62, −1.64	0.98, −1.39

Computer programs: *APEX3* (Bruker, 2014 ▸), *SAINT* (Bruker, 2014 ▸), *SHELXT2014* (Sheldrick, 2015*a* ▸), *SHELXL2018* (Sheldrick, 2015*b* ▸) and *SHELXTL* (Sheldrick, 2008 ▸).

## supplementary crystallographic information

### Bis{3,5-difluoro-2-[4-(2,4,6-trimethylphenyl)pyridin-2-yl]phenyl-κ2N,C1}(picolinato-κ2N,O)iridium(III) (1) Crystal data

**Table:**

[Ir(C_20_H_16_F_2_N)_2_(C_6_H_4_NO_2_)]	*F*(000) = 1848
*M**_r_* = 930.98	*D*_x_ = 1.420 Mg m^−^^3^
Monoclinic, *P*2_1_/*c*	Mo *K*α radiation, λ = 0.71073 Å
*a* = 15.1582 (12) Å	Cell parameters from 9153 reflections
*b* = 12.3530 (9) Å	θ = 2.5–30.7°
*c* = 24.2353 (17) Å	µ = 3.12 mm^−^^1^
β = 106.374 (3)°	*T* = 100 K
*V* = 4354.0 (6) Å^3^	Block, yellow
*Z* = 4	0.12 × 0.12 × 0.12 mm

### Bis{3,5-difluoro-2-[4-(2,4,6-trimethylphenyl)pyridin-2-yl]phenyl-κ2N,C1}(picolinato-κ2N,O)iridium(III) (1) Data collection

**Table:**

Area Bruker PHOTON-II CPAD diffractometer	11535 reflections with *I* > 2σ(*I*)
Radiation source: microfocus	*R*_int_ = 0.042
φ and ω scans	θ_max_ = 30.7°, θ_min_ = 2.2°
Absorption correction: multi-scan (SADABS; Bruker, 2014)	*h* = −21→21
*T*_min_ = 0.576, *T*_max_ = 0.746	*k* = −17→17
74054 measured reflections	*l* = −30→34
13464 independent reflections	

### Bis{3,5-difluoro-2-[4-(2,4,6-trimethylphenyl)pyridin-2-yl]phenyl-κ2N,C1}(picolinato-κ2N,O)iridium(III) (1) Refinement

**Table:**

Refinement on *F*^2^	0 restraints
Least-squares matrix: full	Hydrogen site location: inferred from neighbouring sites
*R*[*F*^2^ > 2σ(*F*^2^)] = 0.030	H-atom parameters constrained
*wR*(*F*^2^) = 0.063	*w* = 1/[σ^2^(*F*_o_^2^) + (0.0107*P*)^2^ + 8.8889*P*] where *P* = (*F*_o_^2^ + 2*F*_c_^2^)/3
*S* = 1.10	(Δ/σ)_max_ = 0.006
13464 reflections	Δρ_max_ = 2.62 e Å^−^^3^
512 parameters	Δρ_min_ = −1.64 e Å^−^^3^

### Bis{3,5-difluoro-2-[4-(2,4,6-trimethylphenyl)pyridin-2-yl]phenyl-κ2N,C1}(picolinato-κ2N,O)iridium(III) (1) Special details

**Table:**

Geometry. All esds (except the esd in the dihedral angle between two l.s. planes) are estimated using the full covariance matrix. The cell esds are taken into account individually in the estimation of esds in distances, angles and torsion angles; correlations between esds in cell parameters are only used when they are defined by crystal symmetry. An approximate (isotropic) treatment of cell esds is used for estimating esds involving l.s. planes.

### Bis{3,5-difluoro-2-[4-(2,4,6-trimethylphenyl)pyridin-2-yl]phenyl-κ2N,C1}(picolinato-κ2N,O)iridium(III) (1) Fractional atomic coordinates and isotropic or equivalent isotropic displacement parameters (Å^2^)

**Table:**

	*x*	*y*	*z*	*U*_iso_*/*U*_eq_	
Ir1	0.44048 (2)	0.26980 (2)	0.61437 (2)	0.01790 (3)	
C1	0.46242 (17)	0.32003 (19)	0.54086 (10)	0.0173 (4)	
C2	0.39581 (18)	0.3451 (2)	0.48951 (10)	0.0194 (5)	
H2A	0.332635	0.330991	0.485277	0.023*	
C3	0.42336 (18)	0.3906 (2)	0.44514 (10)	0.0205 (5)	
F1	0.35653 (11)	0.42187 (13)	0.39699 (6)	0.0247 (3)	
C4	0.51336 (19)	0.4086 (2)	0.44628 (10)	0.0204 (5)	
H4A	0.529744	0.437693	0.414238	0.025*	
C5	0.57841 (18)	0.3818 (2)	0.49690 (11)	0.0198 (5)	
F2	0.66758 (11)	0.40260 (14)	0.49956 (7)	0.0273 (3)	
C6	0.55632 (18)	0.3387 (2)	0.54451 (10)	0.0189 (5)	
C7	0.62144 (18)	0.3177 (2)	0.60110 (10)	0.0201 (5)	
C8	0.71651 (19)	0.3190 (2)	0.61409 (11)	0.0236 (5)	
H8A	0.744865	0.335431	0.584779	0.028*	
C9	0.77108 (19)	0.2966 (2)	0.66960 (12)	0.0262 (6)	
C10	0.72713 (19)	0.2742 (3)	0.71159 (11)	0.0279 (6)	
H10A	0.762028	0.259416	0.750019	0.033*	
C11	0.63216 (19)	0.2736 (2)	0.69675 (11)	0.0252 (5)	
H11A	0.602878	0.258211	0.725735	0.030*	
N1	0.57912 (15)	0.29390 (17)	0.64278 (9)	0.0188 (4)	
C12	0.8732 (2)	0.2943 (3)	0.68225 (12)	0.0329 (7)	
C13	0.9183 (3)	0.1955 (3)	0.68471 (16)	0.0467 (9)	
C14	1.0147 (3)	0.1968 (4)	0.69527 (19)	0.0615 (12)	
H14A	1.046683	0.129977	0.697781	0.074*	
C15	1.0634 (3)	0.2912 (5)	0.70197 (17)	0.0625 (13)	
C16	1.0171 (3)	0.3869 (4)	0.6987 (2)	0.0671 (13)	
H16A	1.050763	0.452802	0.703217	0.080*	
C17	0.9212 (2)	0.3909 (4)	0.68900 (18)	0.0521 (10)	
C18	0.8665 (3)	0.0910 (4)	0.6755 (3)	0.0792 (16)	
H18A	0.908129	0.031842	0.672623	0.119*	
H18B	0.816111	0.095033	0.639851	0.119*	
H18C	0.841417	0.077452	0.707948	0.119*	
C19	1.1680 (3)	0.2894 (6)	0.7136 (3)	0.099 (2)	
H19A	1.189898	0.362465	0.708880	0.148*	
H19B	1.184515	0.240147	0.686417	0.148*	
H19C	1.196445	0.264378	0.752993	0.148*	
C20	0.8725 (3)	0.4990 (4)	0.6840 (3)	0.0834 (18)	
H20A	0.916920	0.555588	0.701383	0.125*	
H20B	0.824712	0.495448	0.704071	0.125*	
H20C	0.844154	0.515872	0.643344	0.125*	
C21	0.45657 (18)	0.1194 (2)	0.58964 (10)	0.0199 (5)	
C22	0.53758 (19)	0.0587 (2)	0.60259 (11)	0.0228 (5)	
H22A	0.592843	0.087662	0.627112	0.027*	
C23	0.5370 (2)	−0.0434 (2)	0.57965 (11)	0.0244 (5)	
F3	0.61738 (12)	−0.09986 (14)	0.59174 (7)	0.0312 (4)	
C24	0.4596 (2)	−0.0919 (2)	0.54407 (12)	0.0265 (6)	
H24A	0.461298	−0.162441	0.528785	0.032*	
C25	0.3798 (2)	−0.0319 (2)	0.53206 (12)	0.0256 (5)	
F4	0.30307 (12)	−0.07915 (14)	0.49674 (8)	0.0347 (4)	
C26	0.37433 (19)	0.0718 (2)	0.55362 (11)	0.0214 (5)	
C27	0.29216 (18)	0.1404 (2)	0.54264 (11)	0.0216 (5)	
C28	0.20602 (19)	0.1192 (2)	0.50477 (12)	0.0255 (5)	
H28A	0.196735	0.054324	0.482825	0.031*	
C29	0.13299 (19)	0.1916 (2)	0.49849 (12)	0.0264 (5)	
C30	0.14919 (19)	0.2857 (2)	0.53145 (13)	0.0273 (6)	
H30A	0.100800	0.335859	0.529270	0.033*	
C31	0.23655 (19)	0.3057 (2)	0.56749 (12)	0.0242 (5)	
H31A	0.247382	0.371254	0.588827	0.029*	
N2	0.30662 (15)	0.23538 (18)	0.57334 (9)	0.0207 (4)	
C32	0.0424 (2)	0.1647 (2)	0.45704 (13)	0.0290 (6)	
C33	0.0347 (2)	0.1588 (3)	0.39791 (13)	0.0329 (6)	
C34	−0.0469 (2)	0.1216 (3)	0.36043 (14)	0.0386 (7)	
H34A	−0.051883	0.117025	0.320534	0.046*	
C35	−0.1215 (2)	0.0909 (3)	0.37967 (14)	0.0364 (7)	
C36	−0.1140 (2)	0.1033 (2)	0.43762 (14)	0.0333 (7)	
H36A	−0.165574	0.086533	0.451004	0.040*	
C37	−0.0340 (2)	0.1391 (2)	0.47677 (13)	0.0302 (6)	
C38	0.1131 (3)	0.1942 (3)	0.37436 (15)	0.0438 (8)	
H38A	0.089207	0.209260	0.333131	0.066*	
H38B	0.159140	0.136384	0.380381	0.066*	
H38C	0.141607	0.259789	0.394411	0.066*	
C39	−0.2074 (2)	0.0433 (3)	0.33887 (16)	0.0483 (9)	
H39A	−0.193167	0.016486	0.304286	0.072*	
H39B	−0.255023	0.099212	0.328086	0.072*	
H39C	−0.229634	−0.016696	0.357818	0.072*	
C40	−0.0297 (2)	0.1475 (3)	0.53984 (13)	0.0355 (7)	
H40A	0.022382	0.105043	0.562743	0.053*	
H40B	−0.086911	0.119468	0.545677	0.053*	
H40C	−0.021905	0.223488	0.551894	0.053*	
O1	0.41561 (13)	0.43013 (14)	0.64265 (7)	0.0207 (4)	
O2	0.39443 (15)	0.52154 (16)	0.71775 (8)	0.0289 (4)	
C41	0.40599 (17)	0.4372 (2)	0.69325 (11)	0.0203 (5)	
C42	0.40885 (17)	0.3319 (2)	0.72522 (10)	0.0198 (5)	
C43	0.3988 (2)	0.3291 (2)	0.78032 (11)	0.0271 (6)	
H43A	0.391480	0.394281	0.799356	0.033*	
C44	0.3995 (2)	0.2302 (3)	0.80721 (13)	0.0337 (6)	
H44A	0.392203	0.226377	0.844824	0.040*	
C45	0.4111 (2)	0.1365 (2)	0.77822 (12)	0.0305 (6)	
H45A	0.411129	0.067594	0.795591	0.037*	
C46	0.42253 (19)	0.1451 (2)	0.72369 (12)	0.0254 (5)	
H46A	0.431422	0.081050	0.704225	0.031*	
N3	0.42147 (15)	0.24099 (18)	0.69746 (9)	0.0202 (4)	

### Bis{3,5-difluoro-2-[4-(2,4,6-trimethylphenyl)pyridin-2-yl]phenyl-κ2N,C1}(picolinato-κ2N,O)iridium(III) (1) Atomic displacement parameters (Å^2^)

**Table:**

	*U*^11^	*U*^22^	*U*^33^	*U*^12^	*U*^13^	*U*^23^
Ir1	0.02447 (5)	0.01372 (4)	0.01498 (4)	−0.00063 (4)	0.00470 (3)	0.00034 (4)
C1	0.0281 (12)	0.0089 (10)	0.0156 (10)	−0.0001 (9)	0.0073 (9)	−0.0003 (8)
C2	0.0256 (12)	0.0123 (11)	0.0188 (11)	−0.0008 (9)	0.0040 (9)	−0.0030 (8)
C3	0.0308 (13)	0.0120 (11)	0.0162 (11)	0.0032 (9)	0.0028 (9)	−0.0031 (8)
F1	0.0314 (8)	0.0233 (8)	0.0167 (7)	0.0049 (6)	0.0024 (6)	0.0025 (6)
C4	0.0334 (14)	0.0125 (11)	0.0163 (11)	0.0016 (10)	0.0084 (10)	−0.0002 (8)
C5	0.0254 (12)	0.0138 (11)	0.0210 (12)	0.0003 (9)	0.0075 (9)	−0.0021 (9)
F2	0.0279 (8)	0.0294 (9)	0.0253 (8)	0.0004 (7)	0.0088 (6)	0.0032 (6)
C6	0.0268 (12)	0.0126 (11)	0.0161 (11)	0.0005 (9)	0.0041 (9)	−0.0019 (8)
C7	0.0280 (13)	0.0115 (11)	0.0189 (11)	0.0009 (9)	0.0037 (9)	−0.0008 (8)
C8	0.0281 (13)	0.0198 (13)	0.0221 (12)	0.0026 (10)	0.0060 (10)	0.0015 (10)
C9	0.0260 (13)	0.0234 (14)	0.0254 (13)	0.0031 (10)	0.0012 (10)	−0.0017 (10)
C10	0.0295 (13)	0.0304 (14)	0.0187 (12)	0.0047 (12)	−0.0015 (10)	0.0015 (11)
C11	0.0311 (13)	0.0252 (13)	0.0177 (11)	−0.0015 (11)	0.0044 (10)	−0.0005 (10)
N1	0.0229 (10)	0.0149 (10)	0.0169 (9)	0.0013 (8)	0.0029 (8)	0.0001 (7)
C12	0.0240 (13)	0.048 (2)	0.0226 (13)	0.0070 (12)	−0.0004 (10)	0.0045 (12)
C13	0.0387 (19)	0.055 (2)	0.049 (2)	0.0164 (17)	0.0151 (16)	0.0065 (17)
C14	0.041 (2)	0.084 (3)	0.059 (3)	0.029 (2)	0.0128 (18)	0.013 (2)
C15	0.0294 (18)	0.110 (4)	0.043 (2)	0.012 (2)	0.0014 (15)	0.011 (2)
C16	0.034 (2)	0.082 (4)	0.073 (3)	−0.011 (2)	−0.0059 (19)	0.007 (3)
C17	0.0271 (17)	0.060 (3)	0.058 (2)	−0.0040 (16)	−0.0064 (15)	0.0019 (19)
C18	0.065 (3)	0.044 (3)	0.134 (5)	0.027 (2)	0.037 (3)	0.003 (3)
C19	0.032 (2)	0.171 (7)	0.086 (4)	0.017 (3)	0.006 (2)	0.030 (4)
C20	0.047 (3)	0.045 (3)	0.140 (5)	−0.017 (2)	−0.003 (3)	−0.003 (3)
C21	0.0295 (13)	0.0172 (12)	0.0141 (11)	−0.0012 (10)	0.0080 (9)	0.0037 (8)
C22	0.0312 (13)	0.0175 (12)	0.0188 (12)	0.0012 (10)	0.0057 (10)	0.0031 (9)
C23	0.0338 (14)	0.0178 (12)	0.0229 (12)	0.0053 (10)	0.0102 (11)	0.0041 (9)
F3	0.0392 (10)	0.0225 (9)	0.0320 (9)	0.0117 (7)	0.0100 (7)	0.0033 (7)
C24	0.0418 (16)	0.0132 (12)	0.0272 (13)	−0.0027 (11)	0.0140 (12)	−0.0018 (10)
C25	0.0335 (14)	0.0185 (13)	0.0247 (13)	−0.0057 (11)	0.0080 (11)	−0.0021 (10)
F4	0.0357 (10)	0.0211 (9)	0.0444 (10)	−0.0075 (7)	0.0065 (8)	−0.0116 (7)
C26	0.0304 (13)	0.0135 (11)	0.0209 (12)	−0.0019 (10)	0.0082 (10)	0.0014 (9)
C27	0.0273 (13)	0.0156 (12)	0.0228 (12)	−0.0024 (10)	0.0084 (10)	0.0018 (9)
C28	0.0316 (14)	0.0185 (13)	0.0246 (13)	−0.0053 (11)	0.0051 (10)	−0.0012 (10)
C29	0.0276 (13)	0.0228 (13)	0.0273 (13)	−0.0035 (11)	0.0051 (10)	0.0046 (11)
C30	0.0245 (13)	0.0200 (14)	0.0360 (15)	0.0012 (10)	0.0065 (11)	0.0020 (11)
C31	0.0290 (13)	0.0180 (12)	0.0271 (13)	−0.0012 (10)	0.0104 (10)	0.0004 (10)
N2	0.0257 (10)	0.0152 (9)	0.0213 (10)	−0.0024 (9)	0.0067 (8)	0.0017 (8)
C32	0.0292 (14)	0.0213 (14)	0.0323 (15)	−0.0022 (11)	0.0019 (11)	0.0042 (11)
C33	0.0357 (16)	0.0237 (14)	0.0353 (16)	−0.0007 (12)	0.0036 (12)	0.0036 (12)
C34	0.0440 (18)	0.0295 (17)	0.0333 (16)	0.0027 (14)	−0.0036 (13)	0.0032 (13)
C35	0.0317 (16)	0.0266 (16)	0.0410 (17)	0.0003 (12)	−0.0061 (13)	0.0037 (13)
C36	0.0246 (14)	0.0242 (15)	0.0448 (18)	−0.0016 (11)	−0.0007 (12)	0.0071 (12)
C37	0.0286 (14)	0.0227 (14)	0.0360 (15)	−0.0001 (11)	0.0040 (11)	0.0071 (11)
C38	0.053 (2)	0.044 (2)	0.0325 (17)	−0.0121 (17)	0.0089 (15)	0.0014 (14)
C39	0.0387 (19)	0.041 (2)	0.051 (2)	−0.0039 (15)	−0.0115 (15)	−0.0008 (16)
C40	0.0303 (15)	0.0360 (18)	0.0374 (17)	−0.0040 (13)	0.0052 (12)	0.0049 (13)
O1	0.0297 (10)	0.0143 (8)	0.0185 (8)	−0.0007 (7)	0.0075 (7)	−0.0003 (6)
O2	0.0448 (12)	0.0199 (10)	0.0237 (10)	0.0001 (9)	0.0122 (9)	−0.0034 (7)
C41	0.0211 (12)	0.0188 (12)	0.0203 (12)	−0.0038 (9)	0.0048 (9)	−0.0008 (9)
C42	0.0203 (11)	0.0200 (13)	0.0184 (11)	0.0000 (9)	0.0042 (9)	0.0009 (9)
C43	0.0359 (15)	0.0274 (15)	0.0198 (12)	0.0032 (12)	0.0107 (11)	0.0021 (10)
C44	0.0468 (17)	0.0340 (16)	0.0241 (13)	0.0067 (14)	0.0163 (12)	0.0077 (12)
C45	0.0392 (16)	0.0256 (15)	0.0279 (14)	0.0057 (12)	0.0113 (12)	0.0128 (11)
C46	0.0320 (14)	0.0195 (13)	0.0255 (13)	0.0035 (11)	0.0094 (11)	0.0033 (10)
N3	0.0243 (10)	0.0178 (11)	0.0179 (9)	−0.0002 (8)	0.0049 (8)	0.0029 (8)

### Bis{3,5-difluoro-2-[4-(2,4,6-trimethylphenyl)pyridin-2-yl]phenyl-κ2N,C1}(picolinato-κ2N,O)iridium(III) (1) Geometric parameters (Å, º)

**Table:**

Ir1—C21	1.988 (3)	C23—F3	1.363 (3)
Ir1—C1	2.001 (2)	C23—C24	1.381 (4)
Ir1—N2	2.037 (2)	C24—C25	1.378 (4)
Ir1—N1	2.040 (2)	C24—H24A	0.9500
Ir1—N3	2.143 (2)	C25—F4	1.365 (3)
Ir1—O1	2.1633 (18)	C25—C26	1.395 (4)
C1—C2	1.398 (3)	C26—C27	1.468 (4)
C1—C6	1.420 (4)	C27—N2	1.373 (3)
C2—C3	1.378 (4)	C27—C28	1.393 (4)
C2—H2A	0.9500	C28—C29	1.398 (4)
C3—F1	1.368 (3)	C28—H28A	0.9500
C3—C4	1.375 (4)	C29—C30	1.392 (4)
C4—C5	1.380 (3)	C29—C32	1.493 (4)
C4—H4A	0.9500	C30—C31	1.388 (4)
C5—F2	1.359 (3)	C30—H30A	0.9500
C5—C6	1.394 (3)	C31—N2	1.348 (4)
C6—C7	1.470 (3)	C31—H31A	0.9500
C7—N1	1.372 (3)	C32—C33	1.407 (4)
C7—C8	1.386 (4)	C32—C37	1.408 (4)
C8—C9	1.394 (4)	C33—C34	1.390 (4)
C8—H8A	0.9500	C33—C38	1.521 (5)
C9—C10	1.392 (4)	C34—C35	1.392 (5)
C9—C12	1.491 (4)	C34—H34A	0.9500
C10—C11	1.382 (4)	C35—C36	1.385 (5)
C10—H10A	0.9500	C35—C39	1.514 (4)
C11—N1	1.352 (3)	C36—C37	1.385 (4)
C11—H11A	0.9500	C36—H36A	0.9500
C12—C17	1.383 (5)	C37—C40	1.515 (4)
C12—C13	1.392 (5)	C38—H38A	0.9800
C13—C14	1.411 (5)	C38—H38B	0.9800
C13—C18	1.495 (6)	C38—H38C	0.9800
C14—C15	1.365 (7)	C39—H39A	0.9800
C14—H14A	0.9500	C39—H39B	0.9800
C15—C16	1.366 (7)	C39—H39C	0.9800
C15—C19	1.529 (6)	C40—H40A	0.9800
C16—C17	1.406 (5)	C40—H40B	0.9800
C16—H16A	0.9500	C40—H40C	0.9800
C17—C20	1.514 (6)	O1—C41	1.278 (3)
C18—H18A	0.9800	O2—C41	1.236 (3)
C18—H18B	0.9800	C41—C42	1.508 (4)
C18—H18C	0.9800	C42—N3	1.350 (3)
C19—H19A	0.9800	C42—C43	1.387 (4)
C19—H19B	0.9800	C43—C44	1.383 (4)
C19—H19C	0.9800	C43—H43A	0.9500
C20—H20A	0.9800	C44—C45	1.390 (4)
C20—H20B	0.9800	C44—H44A	0.9500
C20—H20C	0.9800	C45—C46	1.384 (4)
C21—C22	1.397 (4)	C45—H45A	0.9500
C21—C26	1.431 (4)	C46—N3	1.342 (3)
C22—C23	1.377 (4)	C46—H46A	0.9500
C22—H22A	0.9500		
			
C21—Ir1—C1	87.48 (9)	C23—C22—H22A	120.2
C21—Ir1—N2	81.24 (10)	C21—C22—H22A	120.2
C1—Ir1—N2	91.30 (9)	F3—C23—C22	118.5 (3)
C21—Ir1—N1	92.06 (10)	F3—C23—C24	117.6 (2)
C1—Ir1—N1	80.87 (9)	C22—C23—C24	123.9 (3)
N2—Ir1—N1	169.95 (8)	C25—C24—C23	116.0 (3)
C21—Ir1—N3	100.86 (9)	C25—C24—H24A	122.0
C1—Ir1—N3	171.25 (9)	C23—C24—H24A	122.0
N2—Ir1—N3	92.56 (8)	F4—C25—C24	116.4 (2)
N1—Ir1—N3	96.07 (8)	F4—C25—C26	119.8 (3)
C21—Ir1—O1	176.51 (9)	C24—C25—C26	123.8 (3)
C1—Ir1—O1	94.92 (8)	C25—C26—C21	118.2 (2)
N2—Ir1—O1	96.15 (8)	C25—C26—C27	126.8 (2)
N1—Ir1—O1	90.83 (8)	C21—C26—C27	115.0 (2)
N3—Ir1—O1	76.87 (7)	N2—C27—C28	119.6 (2)
C2—C1—C6	118.6 (2)	N2—C27—C26	113.4 (2)
C2—C1—Ir1	126.97 (19)	C28—C27—C26	126.9 (2)
C6—C1—Ir1	114.22 (17)	C27—C28—C29	121.2 (3)
C3—C2—C1	118.9 (2)	C27—C28—H28A	119.4
C3—C2—H2A	120.6	C29—C28—H28A	119.4
C1—C2—H2A	120.6	C30—C29—C28	117.8 (3)
F1—C3—C4	117.6 (2)	C30—C29—C32	123.5 (3)
F1—C3—C2	117.8 (2)	C28—C29—C32	118.7 (3)
C4—C3—C2	124.6 (2)	C31—C30—C29	119.5 (3)
C3—C4—C5	115.8 (2)	C31—C30—H30A	120.3
C3—C4—H4A	122.1	C29—C30—H30A	120.3
C5—C4—H4A	122.1	N2—C31—C30	122.4 (3)
F2—C5—C4	116.6 (2)	N2—C31—H31A	118.8
F2—C5—C6	120.0 (2)	C30—C31—H31A	118.8
C4—C5—C6	123.3 (2)	C31—N2—C27	119.5 (2)
C5—C6—C1	118.7 (2)	C31—N2—Ir1	124.31 (19)
C5—C6—C7	125.8 (2)	C27—N2—Ir1	115.32 (17)
C1—C6—C7	115.3 (2)	C33—C32—C37	119.7 (3)
N1—C7—C8	120.5 (2)	C33—C32—C29	119.6 (3)
N1—C7—C6	113.2 (2)	C37—C32—C29	120.7 (3)
C8—C7—C6	126.3 (2)	C34—C33—C32	119.1 (3)
C7—C8—C9	120.9 (3)	C34—C33—C38	119.7 (3)
C7—C8—H8A	119.6	C32—C33—C38	121.2 (3)
C9—C8—H8A	119.6	C33—C34—C35	121.9 (3)
C10—C9—C8	118.0 (3)	C33—C34—H34A	119.0
C10—C9—C12	121.9 (2)	C35—C34—H34A	119.0
C8—C9—C12	120.1 (3)	C36—C35—C34	117.8 (3)
C11—C10—C9	119.3 (2)	C36—C35—C39	121.0 (3)
C11—C10—H10A	120.4	C34—C35—C39	121.2 (3)
C9—C10—H10A	120.4	C37—C36—C35	122.4 (3)
N1—C11—C10	122.9 (3)	C37—C36—H36A	118.8
N1—C11—H11A	118.6	C35—C36—H36A	118.8
C10—C11—H11A	118.6	C36—C37—C32	118.9 (3)
C11—N1—C7	118.6 (2)	C36—C37—C40	119.6 (3)
C11—N1—Ir1	125.07 (18)	C32—C37—C40	121.5 (3)
C7—N1—Ir1	115.69 (16)	C33—C38—H38A	109.5
C17—C12—C13	121.0 (3)	C33—C38—H38B	109.5
C17—C12—C9	119.3 (3)	H38A—C38—H38B	109.5
C13—C12—C9	119.6 (3)	C33—C38—H38C	109.5
C12—C13—C14	118.0 (4)	H38A—C38—H38C	109.5
C12—C13—C18	121.4 (3)	H38B—C38—H38C	109.5
C14—C13—C18	120.6 (4)	C35—C39—H39A	109.5
C15—C14—C13	121.9 (4)	C35—C39—H39B	109.5
C15—C14—H14A	119.0	H39A—C39—H39B	109.5
C13—C14—H14A	119.0	C35—C39—H39C	109.5
C14—C15—C16	118.8 (4)	H39A—C39—H39C	109.5
C14—C15—C19	120.5 (5)	H39B—C39—H39C	109.5
C16—C15—C19	120.8 (5)	C37—C40—H40A	109.5
C15—C16—C17	122.0 (5)	C37—C40—H40B	109.5
C15—C16—H16A	119.0	H40A—C40—H40B	109.5
C17—C16—H16A	119.0	C37—C40—H40C	109.5
C12—C17—C16	118.3 (4)	H40A—C40—H40C	109.5
C12—C17—C20	121.5 (3)	H40B—C40—H40C	109.5
C16—C17—C20	120.2 (4)	C41—O1—Ir1	116.32 (16)
C13—C18—H18A	109.5	O2—C41—O1	126.0 (2)
C13—C18—H18B	109.5	O2—C41—C42	117.9 (2)
H18A—C18—H18B	109.5	O1—C41—C42	116.1 (2)
C13—C18—H18C	109.5	N3—C42—C43	121.9 (2)
H18A—C18—H18C	109.5	N3—C42—C41	116.7 (2)
H18B—C18—H18C	109.5	C43—C42—C41	121.4 (2)
C15—C19—H19A	109.5	C44—C43—C42	119.2 (3)
C15—C19—H19B	109.5	C44—C43—H43A	120.4
H19A—C19—H19B	109.5	C42—C43—H43A	120.4
C15—C19—H19C	109.5	C43—C44—C45	118.8 (3)
H19A—C19—H19C	109.5	C43—C44—H44A	120.6
H19B—C19—H19C	109.5	C45—C44—H44A	120.6
C17—C20—H20A	109.5	C46—C45—C44	119.1 (3)
C17—C20—H20B	109.5	C46—C45—H45A	120.4
H20A—C20—H20B	109.5	C44—C45—H45A	120.4
C17—C20—H20C	109.5	N3—C46—C45	122.1 (3)
H20A—C20—H20C	109.5	N3—C46—H46A	118.9
H20B—C20—H20C	109.5	C45—C46—H46A	118.9
C22—C21—C26	118.4 (2)	C46—N3—C42	118.8 (2)
C22—C21—Ir1	127.5 (2)	C46—N3—Ir1	127.22 (18)
C26—C21—Ir1	114.08 (19)	C42—N3—Ir1	113.94 (16)
C23—C22—C21	119.7 (3)		
			
C6—C1—C2—C3	−2.3 (3)	C24—C25—C26—C21	0.9 (4)
Ir1—C1—C2—C3	172.49 (18)	F4—C25—C26—C27	0.1 (4)
C1—C2—C3—F1	−175.2 (2)	C24—C25—C26—C27	179.8 (3)
C1—C2—C3—C4	3.6 (4)	C22—C21—C26—C25	−1.6 (4)
F1—C3—C4—C5	176.3 (2)	Ir1—C21—C26—C25	177.24 (19)
C2—C3—C4—C5	−2.4 (4)	C22—C21—C26—C27	179.3 (2)
C3—C4—C5—F2	−177.7 (2)	Ir1—C21—C26—C27	−1.8 (3)
C3—C4—C5—C6	0.2 (4)	C25—C26—C27—N2	175.4 (2)
F2—C5—C6—C1	178.7 (2)	C21—C26—C27—N2	−5.7 (3)
C4—C5—C6—C1	0.8 (4)	C25—C26—C27—C28	−6.0 (4)
F2—C5—C6—C7	3.7 (4)	C21—C26—C27—C28	172.9 (2)
C4—C5—C6—C7	−174.2 (2)	N2—C27—C28—C29	−1.7 (4)
C2—C1—C6—C5	0.3 (3)	C26—C27—C28—C29	179.8 (3)
Ir1—C1—C6—C5	−175.16 (18)	C27—C28—C29—C30	−0.1 (4)
C2—C1—C6—C7	175.8 (2)	C27—C28—C29—C32	179.7 (3)
Ir1—C1—C6—C7	0.3 (3)	C28—C29—C30—C31	2.0 (4)
C5—C6—C7—N1	168.8 (2)	C32—C29—C30—C31	−177.9 (3)
C1—C6—C7—N1	−6.3 (3)	C29—C30—C31—N2	−2.1 (4)
C5—C6—C7—C8	−12.0 (4)	C30—C31—N2—C27	0.2 (4)
C1—C6—C7—C8	172.9 (2)	C30—C31—N2—Ir1	169.2 (2)
N1—C7—C8—C9	−0.2 (4)	C28—C27—N2—C31	1.6 (4)
C6—C7—C8—C9	−179.3 (2)	C26—C27—N2—C31	−179.6 (2)
C7—C8—C9—C10	−0.7 (4)	C28—C27—N2—Ir1	−168.25 (19)
C7—C8—C9—C12	177.7 (3)	C26—C27—N2—Ir1	10.5 (3)
C8—C9—C10—C11	0.8 (4)	C30—C29—C32—C33	115.0 (3)
C12—C9—C10—C11	−177.6 (3)	C28—C29—C32—C33	−64.9 (4)
C9—C10—C11—N1	0.0 (5)	C30—C29—C32—C37	−68.7 (4)
C10—C11—N1—C7	−1.0 (4)	C28—C29—C32—C37	111.5 (3)
C10—C11—N1—Ir1	169.1 (2)	C37—C32—C33—C34	−3.7 (5)
C8—C7—N1—C11	1.1 (4)	C29—C32—C33—C34	172.6 (3)
C6—C7—N1—C11	−179.7 (2)	C37—C32—C33—C38	175.1 (3)
C8—C7—N1—Ir1	−169.93 (19)	C29—C32—C33—C38	−8.5 (5)
C6—C7—N1—Ir1	9.3 (3)	C32—C33—C34—C35	0.5 (5)
C10—C9—C12—C17	−103.3 (4)	C38—C33—C34—C35	−178.3 (3)
C8—C9—C12—C17	78.3 (4)	C33—C34—C35—C36	3.0 (5)
C10—C9—C12—C13	79.3 (4)	C33—C34—C35—C39	−175.7 (3)
C8—C9—C12—C13	−99.1 (4)	C34—C35—C36—C37	−3.5 (5)
C17—C12—C13—C14	1.1 (5)	C39—C35—C36—C37	175.2 (3)
C9—C12—C13—C14	178.3 (3)	C35—C36—C37—C32	0.4 (5)
C17—C12—C13—C18	−177.9 (4)	C35—C36—C37—C40	−178.1 (3)
C9—C12—C13—C18	−0.6 (5)	C33—C32—C37—C36	3.3 (4)
C12—C13—C14—C15	−1.1 (6)	C29—C32—C37—C36	−173.1 (3)
C18—C13—C14—C15	177.8 (4)	C33—C32—C37—C40	−178.2 (3)
C13—C14—C15—C16	0.5 (7)	C29—C32—C37—C40	5.5 (4)
C13—C14—C15—C19	179.7 (4)	Ir1—O1—C41—O2	177.5 (2)
C14—C15—C16—C17	0.3 (7)	Ir1—O1—C41—C42	−2.8 (3)
C19—C15—C16—C17	−179.0 (4)	O2—C41—C42—N3	179.9 (2)
C13—C12—C17—C16	−0.3 (6)	O1—C41—C42—N3	0.2 (3)
C9—C12—C17—C16	−177.6 (3)	O2—C41—C42—C43	0.1 (4)
C13—C12—C17—C20	177.5 (4)	O1—C41—C42—C43	−179.7 (2)
C9—C12—C17—C20	0.2 (6)	N3—C42—C43—C44	−1.5 (4)
C15—C16—C17—C12	−0.4 (7)	C41—C42—C43—C44	178.3 (3)
C15—C16—C17—C20	−178.2 (5)	C42—C43—C44—C45	0.5 (5)
C26—C21—C22—C23	1.4 (4)	C43—C44—C45—C46	0.7 (5)
Ir1—C21—C22—C23	−177.24 (19)	C44—C45—C46—N3	−1.0 (5)
C21—C22—C23—F3	178.3 (2)	C45—C46—N3—C42	0.0 (4)
C21—C22—C23—C24	−0.5 (4)	C45—C46—N3—Ir1	178.7 (2)
F3—C23—C24—C25	−179.1 (2)	C43—C42—N3—C46	1.3 (4)
C22—C23—C24—C25	−0.2 (4)	C41—C42—N3—C46	−178.6 (2)
C23—C24—C25—F4	179.8 (2)	C43—C42—N3—Ir1	−177.6 (2)
C23—C24—C25—C26	0.0 (4)	C41—C42—N3—Ir1	2.5 (3)
F4—C25—C26—C21	−178.9 (2)		

### Bis[2-(4-tert-butylpyridin-2-yl)-3,5-difluorophenyl-κ2N,C1](picolinato-κ2N,O)iridium(III) (2) Crystal data

**Table:**

[Ir(C_15_H_14_F_2_N)_2_(C_6_H_4_NO_2_)]	*F*(000) = 1592
*M**_r_* = 806.84	*D*_x_ = 1.406 Mg m^−^^3^
Monoclinic, *P*2_1_/*c*	Mo *K*α radiation, λ = 0.71073 Å
*a* = 14.7519 (7) Å	Cell parameters from 2779 reflections
*b* = 14.4121 (7) Å	θ = 2.8–30.1°
*c* = 18.5425 (8) Å	µ = 3.55 mm^−^^1^
β = 104.7761 (19)°	*T* = 100 K
*V* = 3811.9 (3) Å^3^	Plate, yellow
*Z* = 4	0.15 × 0.13 × 0.04 mm

### Bis[2-(4-tert-butylpyridin-2-yl)-3,5-difluorophenyl-κ2N,C1](picolinato-κ2N,O)iridium(III) (2) Data collection

**Table:**

Area Bruker PHOTON-II CPAD diffractometer	9342 reflections with *I* > 2σ(*I*)
Radiation source: microfocus	*R*_int_ = 0.049
φ and ω scans	θ_max_ = 30.6°, θ_min_ = 2.4°
Absorption correction: multi-scan (SADABS; Bruker, 2014)	*h* = −21→21
*T*_min_ = 0.370, *T*_max_ = 0.433	*k* = −20→20
81284 measured reflections	*l* = −26→19
11662 independent reflections	

### Bis[2-(4-tert-butylpyridin-2-yl)-3,5-difluorophenyl-κ2N,C1](picolinato-κ2N,O)iridium(III) (2) Refinement

**Table:**

Refinement on *F*^2^	0 restraints
Least-squares matrix: full	Hydrogen site location: inferred from neighbouring sites
*R*[*F*^2^ > 2σ(*F*^2^)] = 0.025	H-atom parameters constrained
*wR*(*F*^2^) = 0.057	*w* = 1/[σ^2^(*F*_o_^2^) + (0.0242*P*)^2^ + 2.756*P*] where *P* = (*F*_o_^2^ + 2*F*_c_^2^)/3
*S* = 1.02	(Δ/σ)_max_ = 0.002
11662 reflections	Δρ_max_ = 0.98 e Å^−^^3^
421 parameters	Δρ_min_ = −1.39 e Å^−^^3^

### Bis[2-(4-tert-butylpyridin-2-yl)-3,5-difluorophenyl-κ2N,C1](picolinato-κ2N,O)iridium(III) (2) Special details

**Table:**

Geometry. All esds (except the esd in the dihedral angle between two l.s. planes) are estimated using the full covariance matrix. The cell esds are taken into account individually in the estimation of esds in distances, angles and torsion angles; correlations between esds in cell parameters are only used when they are defined by crystal symmetry. An approximate (isotropic) treatment of cell esds is used for estimating esds involving l.s. planes.

### Bis[2-(4-tert-butylpyridin-2-yl)-3,5-difluorophenyl-κ2N,C1](picolinato-κ2N,O)iridium(III) (2) Fractional atomic coordinates and isotropic or equivalent isotropic displacement parameters (Å^2^)

**Table:**

	*x*	*y*	*z*	*U*_iso_*/*U*_eq_	
Ir1	0.30539 (2)	0.53433 (2)	0.67510 (2)	0.01538 (3)	
C1	0.23415 (16)	0.65492 (15)	0.65931 (13)	0.0171 (4)	
C2	0.17630 (18)	0.68813 (17)	0.59293 (14)	0.0231 (5)	
H2	0.167851	0.653112	0.548305	0.028*	
C3	0.13158 (19)	0.77159 (18)	0.59224 (16)	0.0280 (6)	
F1	0.07501 (14)	0.80352 (12)	0.52723 (10)	0.0479 (5)	
C4	0.13985 (18)	0.82624 (17)	0.65481 (16)	0.0275 (6)	
H4	0.108614	0.884197	0.652714	0.033*	
C5	0.19550 (18)	0.79214 (16)	0.71984 (15)	0.0239 (5)	
F2	0.20190 (12)	0.84419 (11)	0.78251 (9)	0.0348 (4)	
C6	0.24427 (16)	0.70822 (16)	0.72467 (13)	0.0179 (5)	
C7	0.30904 (17)	0.67197 (16)	0.79204 (13)	0.0192 (5)	
C8	0.34074 (18)	0.71680 (17)	0.86029 (14)	0.0227 (5)	
H8	0.314210	0.775053	0.867499	0.027*	
C9	0.41011 (19)	0.67912 (18)	0.91841 (14)	0.0249 (5)	
C10	0.4435 (2)	0.5920 (2)	0.90580 (14)	0.0297 (6)	
H10	0.489611	0.562161	0.943949	0.036*	
C11	0.4095 (2)	0.54871 (18)	0.83770 (14)	0.0266 (6)	
H11	0.432831	0.488843	0.830709	0.032*	
N1	0.34539 (14)	0.58692 (13)	0.78110 (11)	0.0190 (4)	
C12	0.4463 (2)	0.7337 (2)	0.99013 (15)	0.0312 (6)	
C13	0.4881 (2)	0.8263 (2)	0.97229 (17)	0.0392 (7)	
H13A	0.512994	0.860843	1.018614	0.059*	
H13B	0.439081	0.862971	0.938708	0.059*	
H13C	0.538732	0.813863	0.948183	0.059*	
C14	0.5224 (2)	0.6809 (2)	1.04678 (16)	0.0456 (8)	
H14A	0.544789	0.718886	1.091608	0.068*	
H14B	0.574806	0.667242	1.024868	0.068*	
H14C	0.496480	0.622629	1.060121	0.068*	
C15	0.3636 (2)	0.7541 (2)	1.02501 (16)	0.0355 (7)	
H15A	0.385539	0.792902	1.069460	0.053*	
H15B	0.338844	0.695600	1.039034	0.053*	
H15C	0.313999	0.786809	0.988629	0.053*	
C16	0.18532 (17)	0.47689 (15)	0.68282 (14)	0.0198 (5)	
C17	0.15357 (19)	0.46982 (19)	0.74756 (16)	0.0279 (6)	
H17	0.189174	0.494662	0.793538	0.034*	
C18	0.0694 (2)	0.4259 (2)	0.74288 (17)	0.0325 (6)	
F3	0.03862 (13)	0.41878 (15)	0.80583 (10)	0.0486 (5)	
C19	0.0135 (2)	0.3893 (2)	0.67848 (19)	0.0396 (7)	
H19	−0.044204	0.359379	0.677508	0.048*	
C20	0.0456 (2)	0.3983 (2)	0.61536 (18)	0.0351 (7)	
F4	−0.01075 (13)	0.36506 (15)	0.55072 (11)	0.0529 (6)	
C21	0.13079 (19)	0.43920 (17)	0.61503 (15)	0.0242 (5)	
C22	0.17271 (18)	0.44401 (17)	0.55140 (15)	0.0219 (5)	
C23	0.13701 (18)	0.40646 (18)	0.48020 (15)	0.0267 (6)	
H23	0.077070	0.377730	0.468464	0.032*	
C24	0.18727 (17)	0.41028 (17)	0.42637 (14)	0.0224 (5)	
C25	0.27440 (17)	0.45626 (17)	0.44603 (14)	0.0218 (5)	
H25	0.311171	0.461624	0.410983	0.026*	
C26	0.30581 (17)	0.49330 (16)	0.51629 (14)	0.0196 (5)	
H26	0.364411	0.524625	0.528415	0.023*	
N2	0.25766 (14)	0.48739 (13)	0.56872 (11)	0.0177 (4)	
C27	0.15441 (19)	0.36300 (19)	0.35017 (15)	0.0272 (6)	
C28	0.0537 (2)	0.3279 (2)	0.33600 (17)	0.0367 (7)	
H28A	0.034009	0.301118	0.285838	0.055*	
H28B	0.012219	0.379662	0.340076	0.055*	
H28C	0.050216	0.280341	0.372987	0.055*	
C29	0.1604 (2)	0.4314 (2)	0.28779 (16)	0.0347 (7)	
H29A	0.140783	0.400165	0.239377	0.052*	
H29B	0.225128	0.453235	0.295702	0.052*	
H29C	0.119114	0.484491	0.288601	0.052*	
C30	0.2200 (2)	0.2813 (2)	0.34824 (18)	0.0399 (7)	
H30A	0.199692	0.249467	0.300129	0.060*	
H30B	0.218259	0.237926	0.388538	0.060*	
H30C	0.284138	0.304327	0.354822	0.060*	
O1	0.43862 (11)	0.58218 (10)	0.66085 (9)	0.0163 (3)	
O2	0.58841 (12)	0.54150 (12)	0.67624 (11)	0.0254 (4)	
C31	0.50763 (17)	0.52575 (15)	0.67930 (12)	0.0169 (4)	
C32	0.48442 (17)	0.43140 (16)	0.70654 (12)	0.0170 (4)	
C33	0.55271 (19)	0.36654 (17)	0.73644 (14)	0.0227 (5)	
H33	0.616864	0.379203	0.739843	0.027*	
C34	0.5259 (2)	0.28231 (17)	0.76150 (15)	0.0262 (6)	
H34	0.571751	0.237641	0.783980	0.031*	
C35	0.4321 (2)	0.26449 (17)	0.75335 (14)	0.0255 (6)	
H35	0.412206	0.206312	0.767920	0.031*	
C36	0.36739 (19)	0.33247 (16)	0.72366 (13)	0.0224 (5)	
H36	0.302752	0.320269	0.718450	0.027*	
N3	0.39283 (14)	0.41561 (12)	0.70182 (10)	0.0167 (4)	

### Bis[2-(4-tert-butylpyridin-2-yl)-3,5-difluorophenyl-κ2N,C1](picolinato-κ2N,O)iridium(III) (2) Atomic displacement parameters (Å^2^)

**Table:**

	*U*^11^	*U*^22^	*U*^33^	*U*^12^	*U*^13^	*U*^23^
Ir1	0.01640 (5)	0.01245 (4)	0.01887 (4)	0.00043 (4)	0.00740 (3)	−0.00049 (4)
C1	0.0156 (11)	0.0118 (9)	0.0256 (11)	0.0016 (8)	0.0083 (10)	0.0007 (9)
C2	0.0211 (13)	0.0208 (11)	0.0271 (12)	−0.0005 (10)	0.0056 (11)	−0.0017 (10)
C3	0.0196 (13)	0.0263 (13)	0.0346 (15)	0.0043 (10)	0.0003 (11)	0.0020 (11)
F1	0.0514 (12)	0.0343 (9)	0.0438 (11)	0.0202 (9)	−0.0142 (9)	0.0006 (8)
C4	0.0202 (13)	0.0170 (11)	0.0457 (16)	0.0063 (10)	0.0089 (12)	0.0002 (11)
C5	0.0221 (13)	0.0162 (11)	0.0357 (14)	0.0003 (9)	0.0115 (11)	−0.0066 (10)
F2	0.0370 (10)	0.0268 (8)	0.0407 (9)	0.0108 (7)	0.0101 (8)	−0.0110 (7)
C6	0.0137 (11)	0.0167 (10)	0.0251 (12)	0.0005 (8)	0.0081 (10)	−0.0010 (9)
C7	0.0181 (12)	0.0165 (10)	0.0257 (12)	−0.0008 (9)	0.0109 (10)	−0.0012 (9)
C8	0.0278 (14)	0.0192 (11)	0.0244 (12)	−0.0002 (10)	0.0127 (11)	−0.0029 (9)
C9	0.0304 (14)	0.0263 (12)	0.0201 (11)	0.0002 (11)	0.0103 (11)	−0.0033 (10)
C10	0.0365 (16)	0.0336 (14)	0.0179 (11)	0.0100 (12)	0.0049 (11)	0.0000 (11)
C11	0.0354 (15)	0.0236 (13)	0.0230 (12)	0.0100 (11)	0.0113 (11)	−0.0006 (10)
N1	0.0228 (11)	0.0158 (9)	0.0200 (9)	0.0023 (8)	0.0082 (8)	0.0009 (8)
C12	0.0409 (17)	0.0305 (14)	0.0227 (13)	−0.0001 (12)	0.0093 (12)	−0.0070 (11)
C13	0.0423 (19)	0.0450 (17)	0.0319 (15)	−0.0131 (15)	0.0126 (14)	−0.0134 (13)
C14	0.054 (2)	0.052 (2)	0.0246 (14)	0.0127 (17)	−0.0003 (15)	−0.0107 (14)
C15	0.051 (2)	0.0314 (14)	0.0283 (14)	−0.0032 (13)	0.0169 (14)	−0.0121 (12)
C16	0.0175 (11)	0.0149 (10)	0.0297 (12)	−0.0007 (9)	0.0112 (10)	0.0020 (9)
C17	0.0257 (13)	0.0296 (13)	0.0329 (14)	0.0022 (11)	0.0157 (12)	0.0055 (12)
C18	0.0320 (16)	0.0340 (14)	0.0414 (16)	0.0015 (12)	0.0272 (14)	0.0069 (13)
F3	0.0412 (11)	0.0680 (13)	0.0474 (11)	−0.0049 (10)	0.0309 (9)	0.0113 (10)
C19	0.0321 (16)	0.0363 (16)	0.061 (2)	−0.0101 (13)	0.0318 (16)	−0.0054 (15)
C20	0.0263 (15)	0.0348 (15)	0.0487 (17)	−0.0131 (12)	0.0181 (13)	−0.0139 (14)
F4	0.0334 (10)	0.0725 (14)	0.0595 (12)	−0.0292 (10)	0.0243 (9)	−0.0327 (11)
C21	0.0210 (13)	0.0217 (11)	0.0337 (14)	−0.0035 (10)	0.0137 (11)	−0.0054 (10)
C22	0.0177 (12)	0.0202 (11)	0.0292 (13)	−0.0023 (9)	0.0087 (10)	−0.0058 (10)
C23	0.0191 (12)	0.0275 (13)	0.0356 (14)	−0.0090 (10)	0.0105 (11)	−0.0119 (11)
C24	0.0178 (12)	0.0226 (12)	0.0267 (12)	−0.0013 (9)	0.0055 (10)	−0.0086 (10)
C25	0.0189 (12)	0.0236 (12)	0.0251 (12)	−0.0026 (10)	0.0097 (10)	−0.0054 (10)
C26	0.0181 (12)	0.0176 (10)	0.0242 (12)	−0.0021 (9)	0.0075 (10)	−0.0008 (9)
N2	0.0171 (10)	0.0145 (9)	0.0215 (10)	0.0001 (7)	0.0048 (8)	−0.0026 (7)
C27	0.0217 (13)	0.0320 (13)	0.0278 (13)	−0.0047 (11)	0.0059 (11)	−0.0113 (11)
C28	0.0270 (15)	0.0447 (17)	0.0386 (16)	−0.0140 (13)	0.0090 (13)	−0.0186 (14)
C29	0.0302 (16)	0.0440 (16)	0.0288 (14)	−0.0086 (13)	0.0053 (13)	−0.0063 (13)
C30	0.0388 (18)	0.0368 (16)	0.0437 (17)	0.0009 (14)	0.0099 (14)	−0.0194 (14)
O1	0.0149 (8)	0.0132 (7)	0.0213 (8)	−0.0007 (6)	0.0054 (7)	0.0005 (6)
O2	0.0187 (9)	0.0244 (9)	0.0351 (10)	−0.0003 (7)	0.0108 (8)	0.0033 (8)
C31	0.0198 (11)	0.0154 (10)	0.0165 (10)	−0.0001 (9)	0.0062 (9)	−0.0015 (8)
C32	0.0225 (12)	0.0150 (9)	0.0158 (10)	0.0004 (9)	0.0093 (10)	−0.0010 (8)
C33	0.0241 (13)	0.0218 (11)	0.0243 (12)	0.0069 (10)	0.0102 (11)	0.0036 (10)
C34	0.0322 (15)	0.0211 (12)	0.0289 (13)	0.0112 (11)	0.0141 (12)	0.0052 (10)
C35	0.0381 (16)	0.0157 (11)	0.0276 (13)	0.0016 (10)	0.0170 (12)	0.0029 (10)
C36	0.0299 (14)	0.0171 (11)	0.0240 (12)	−0.0028 (10)	0.0137 (11)	−0.0017 (9)
N3	0.0223 (10)	0.0119 (8)	0.0185 (9)	0.0023 (7)	0.0098 (8)	−0.0010 (7)

### Bis[2-(4-tert-butylpyridin-2-yl)-3,5-difluorophenyl-κ2N,C1](picolinato-κ2N,O)iridium(III) (2) Geometric parameters (Å, º)

**Table:**

Ir1—C16	1.993 (2)	C18—F3	1.360 (3)
Ir1—C1	2.013 (2)	C18—C19	1.372 (4)
Ir1—N2	2.034 (2)	C19—C20	1.376 (4)
Ir1—N1	2.048 (2)	C19—H19	0.9500
Ir1—N3	2.1238 (19)	C20—F4	1.360 (3)
Ir1—O1	2.1617 (16)	C20—C21	1.389 (4)
C1—C2	1.392 (3)	C21—C22	1.467 (4)
C1—C6	1.410 (3)	C22—N2	1.363 (3)
C2—C3	1.370 (3)	C22—C23	1.399 (4)
C2—H2	0.9500	C23—C24	1.388 (3)
C3—F1	1.360 (3)	C23—H23	0.9500
C3—C4	1.382 (4)	C24—C25	1.409 (3)
C4—C5	1.365 (4)	C24—C27	1.532 (3)
C4—H4	0.9500	C25—C26	1.374 (3)
C5—F2	1.366 (3)	C25—H25	0.9500
C5—C6	1.398 (3)	C26—N2	1.345 (3)
C6—C7	1.462 (3)	C26—H26	0.9500
C7—N1	1.374 (3)	C27—C28	1.528 (4)
C7—C8	1.391 (3)	C27—C30	1.530 (4)
C8—C9	1.393 (4)	C27—C29	1.540 (4)
C8—H8	0.9500	C28—H28A	0.9800
C9—C10	1.391 (4)	C28—H28B	0.9800
C9—C12	1.520 (4)	C28—H28C	0.9800
C10—C11	1.382 (4)	C29—H29A	0.9800
C10—H10	0.9500	C29—H29B	0.9800
C11—N1	1.339 (3)	C29—H29C	0.9800
C11—H11	0.9500	C30—H30A	0.9800
C12—C14	1.530 (4)	C30—H30B	0.9800
C12—C13	1.541 (4)	C30—H30C	0.9800
C12—C15	1.548 (4)	O1—C31	1.280 (3)
C13—H13A	0.9800	O2—C31	1.229 (3)
C13—H13B	0.9800	C31—C32	1.520 (3)
C13—H13C	0.9800	C32—N3	1.351 (3)
C14—H14A	0.9800	C32—C33	1.382 (3)
C14—H14B	0.9800	C33—C34	1.393 (3)
C14—H14C	0.9800	C33—H33	0.9500
C15—H15A	0.9800	C34—C35	1.377 (4)
C15—H15B	0.9800	C34—H34	0.9500
C15—H15C	0.9800	C35—C36	1.380 (4)
C16—C17	1.399 (3)	C35—H35	0.9500
C16—C21	1.416 (4)	C36—N3	1.348 (3)
C17—C18	1.376 (4)	C36—H36	0.9500
C17—H17	0.9500		
			
C16—Ir1—C1	85.90 (9)	C18—C17—H17	120.9
C16—Ir1—N2	80.63 (9)	C16—C17—H17	120.9
C1—Ir1—N2	96.16 (9)	F3—C18—C19	117.4 (3)
C16—Ir1—N1	97.39 (9)	F3—C18—C17	118.3 (3)
C1—Ir1—N1	80.33 (9)	C19—C18—C17	124.3 (3)
N2—Ir1—N1	176.12 (8)	C18—C19—C20	116.3 (3)
C16—Ir1—N3	98.43 (8)	C18—C19—H19	121.9
C1—Ir1—N3	173.10 (9)	C20—C19—H19	121.9
N2—Ir1—N3	89.87 (7)	F4—C20—C19	116.8 (3)
N1—Ir1—N3	93.73 (7)	F4—C20—C21	119.7 (3)
C16—Ir1—O1	173.52 (8)	C19—C20—C21	123.5 (3)
C1—Ir1—O1	99.44 (8)	C20—C21—C16	117.9 (2)
N2—Ir1—O1	95.06 (7)	C20—C21—C22	126.2 (3)
N1—Ir1—O1	87.18 (7)	C16—C21—C22	115.8 (2)
N3—Ir1—O1	76.61 (7)	N2—C22—C23	120.1 (2)
C2—C1—C6	118.6 (2)	N2—C22—C21	112.5 (2)
C2—C1—Ir1	127.24 (18)	C23—C22—C21	127.3 (2)
C6—C1—Ir1	114.10 (17)	C24—C23—C22	121.4 (2)
C3—C2—C1	119.7 (2)	C24—C23—H23	119.3
C3—C2—H2	120.2	C22—C23—H23	119.3
C1—C2—H2	120.2	C23—C24—C25	116.9 (2)
F1—C3—C2	119.4 (2)	C23—C24—C27	123.0 (2)
F1—C3—C4	117.0 (2)	C25—C24—C27	120.1 (2)
C2—C3—C4	123.6 (3)	C26—C25—C24	119.5 (2)
C5—C4—C3	116.2 (2)	C26—C25—H25	120.2
C5—C4—H4	121.9	C24—C25—H25	120.2
C3—C4—H4	121.9	N2—C26—C25	123.2 (2)
C4—C5—F2	116.7 (2)	N2—C26—H26	118.4
C4—C5—C6	123.5 (2)	C25—C26—H26	118.4
F2—C5—C6	119.8 (2)	C26—N2—C22	118.8 (2)
C5—C6—C1	118.4 (2)	C26—N2—Ir1	124.33 (17)
C5—C6—C7	125.3 (2)	C22—N2—Ir1	116.81 (16)
C1—C6—C7	116.3 (2)	C28—C27—C30	109.5 (2)
N1—C7—C8	119.7 (2)	C28—C27—C24	111.8 (2)
N1—C7—C6	112.8 (2)	C30—C27—C24	108.1 (2)
C8—C7—C6	127.3 (2)	C28—C27—C29	108.8 (2)
C7—C8—C9	122.2 (2)	C30—C27—C29	108.5 (2)
C7—C8—H8	118.9	C24—C27—C29	110.0 (2)
C9—C8—H8	118.9	C27—C28—H28A	109.5
C10—C9—C8	116.3 (2)	C27—C28—H28B	109.5
C10—C9—C12	123.6 (2)	H28A—C28—H28B	109.5
C8—C9—C12	120.0 (2)	C27—C28—H28C	109.5
C11—C10—C9	120.0 (2)	H28A—C28—H28C	109.5
C11—C10—H10	120.0	H28B—C28—H28C	109.5
C9—C10—H10	120.0	C27—C29—H29A	109.5
N1—C11—C10	123.3 (2)	C27—C29—H29B	109.5
N1—C11—H11	118.3	H29A—C29—H29B	109.5
C10—C11—H11	118.3	C27—C29—H29C	109.5
C11—N1—C7	118.4 (2)	H29A—C29—H29C	109.5
C11—N1—Ir1	124.96 (16)	H29B—C29—H29C	109.5
C7—N1—Ir1	116.37 (16)	C27—C30—H30A	109.5
C9—C12—C14	112.1 (2)	C27—C30—H30B	109.5
C9—C12—C13	109.3 (2)	H30A—C30—H30B	109.5
C14—C12—C13	108.3 (3)	C27—C30—H30C	109.5
C9—C12—C15	109.0 (2)	H30A—C30—H30C	109.5
C14—C12—C15	109.1 (2)	H30B—C30—H30C	109.5
C13—C12—C15	109.0 (2)	C31—O1—Ir1	116.79 (14)
C12—C13—H13A	109.5	O2—C31—O1	125.8 (2)
C12—C13—H13B	109.5	O2—C31—C32	119.0 (2)
H13A—C13—H13B	109.5	O1—C31—C32	115.2 (2)
C12—C13—H13C	109.5	N3—C32—C33	121.8 (2)
H13A—C13—H13C	109.5	N3—C32—C31	115.8 (2)
H13B—C13—H13C	109.5	C33—C32—C31	122.4 (2)
C12—C14—H14A	109.5	C32—C33—C34	118.9 (2)
C12—C14—H14B	109.5	C32—C33—H33	120.6
H14A—C14—H14B	109.5	C34—C33—H33	120.6
C12—C14—H14C	109.5	C35—C34—C33	119.3 (2)
H14A—C14—H14C	109.5	C35—C34—H34	120.4
H14B—C14—H14C	109.5	C33—C34—H34	120.4
C12—C15—H15A	109.5	C34—C35—C36	119.0 (2)
C12—C15—H15B	109.5	C34—C35—H35	120.5
H15A—C15—H15B	109.5	C36—C35—H35	120.5
C12—C15—H15C	109.5	N3—C36—C35	122.2 (2)
H15A—C15—H15C	109.5	N3—C36—H36	118.9
H15B—C15—H15C	109.5	C35—C36—H36	118.9
C17—C16—C21	119.7 (2)	C36—N3—C32	118.7 (2)
C17—C16—Ir1	126.2 (2)	C36—N3—Ir1	125.94 (17)
C21—C16—Ir1	114.18 (17)	C32—N3—Ir1	114.67 (14)
C18—C17—C16	118.3 (3)		
			
C6—C1—C2—C3	0.7 (4)	C19—C20—C21—C16	−2.7 (5)
Ir1—C1—C2—C3	178.94 (19)	F4—C20—C21—C22	−5.8 (5)
C1—C2—C3—F1	−179.9 (2)	C19—C20—C21—C22	174.5 (3)
C1—C2—C3—C4	−0.4 (4)	C17—C16—C21—C20	1.6 (4)
F1—C3—C4—C5	178.8 (2)	Ir1—C16—C21—C20	−179.6 (2)
C2—C3—C4—C5	−0.7 (4)	C17—C16—C21—C22	−175.8 (2)
C3—C4—C5—F2	−178.0 (2)	Ir1—C16—C21—C22	2.9 (3)
C3—C4—C5—C6	1.6 (4)	C20—C21—C22—N2	−179.0 (3)
C4—C5—C6—C1	−1.3 (4)	C16—C21—C22—N2	−1.8 (3)
F2—C5—C6—C1	178.3 (2)	C20—C21—C22—C23	−2.1 (5)
C4—C5—C6—C7	176.0 (2)	C16—C21—C22—C23	175.2 (3)
F2—C5—C6—C7	−4.4 (4)	N2—C22—C23—C24	1.5 (4)
C2—C1—C6—C5	0.1 (3)	C21—C22—C23—C24	−175.3 (3)
Ir1—C1—C6—C5	−178.36 (18)	C22—C23—C24—C25	−2.0 (4)
C2—C1—C6—C7	−177.4 (2)	C22—C23—C24—C27	174.7 (2)
Ir1—C1—C6—C7	4.1 (3)	C23—C24—C25—C26	0.9 (4)
C5—C6—C7—N1	178.6 (2)	C27—C24—C25—C26	−175.9 (2)
C1—C6—C7—N1	−4.1 (3)	C24—C25—C26—N2	0.6 (4)
C5—C6—C7—C8	−6.7 (4)	C25—C26—N2—C22	−1.2 (4)
C1—C6—C7—C8	170.6 (2)	C25—C26—N2—Ir1	176.09 (18)
N1—C7—C8—C9	1.2 (4)	C23—C22—N2—C26	0.1 (4)
C6—C7—C8—C9	−173.2 (2)	C21—C22—N2—C26	177.3 (2)
C7—C8—C9—C10	−2.7 (4)	C23—C22—N2—Ir1	−177.36 (19)
C7—C8—C9—C12	176.3 (2)	C21—C22—N2—Ir1	−0.1 (3)
C8—C9—C10—C11	1.7 (4)	C23—C24—C27—C28	10.5 (4)
C12—C9—C10—C11	−177.3 (3)	C25—C24—C27—C28	−172.9 (3)
C9—C10—C11—N1	0.8 (4)	C23—C24—C27—C30	−110.1 (3)
C10—C11—N1—C7	−2.4 (4)	C25—C24—C27—C30	66.5 (3)
C10—C11—N1—Ir1	171.5 (2)	C23—C24—C27—C29	131.5 (3)
C8—C7—N1—C11	1.4 (3)	C25—C24—C27—C29	−51.9 (3)
C6—C7—N1—C11	176.5 (2)	Ir1—O1—C31—O2	−178.69 (19)
C8—C7—N1—Ir1	−173.01 (18)	Ir1—O1—C31—C32	1.8 (2)
C6—C7—N1—Ir1	2.1 (3)	O2—C31—C32—N3	−173.9 (2)
C10—C9—C12—C14	−0.8 (4)	O1—C31—C32—N3	5.6 (3)
C8—C9—C12—C14	−179.7 (3)	O2—C31—C32—C33	7.9 (3)
C10—C9—C12—C13	119.3 (3)	O1—C31—C32—C33	−172.5 (2)
C8—C9—C12—C13	−59.6 (3)	N3—C32—C33—C34	0.8 (4)
C10—C9—C12—C15	−121.6 (3)	C31—C32—C33—C34	178.8 (2)
C8—C9—C12—C15	59.5 (3)	C32—C33—C34—C35	2.4 (4)
C21—C16—C17—C18	0.0 (4)	C33—C34—C35—C36	−3.0 (4)
Ir1—C16—C17—C18	−178.6 (2)	C34—C35—C36—N3	0.5 (4)
C16—C17—C18—F3	179.8 (2)	C35—C36—N3—C32	2.6 (3)
C16—C17—C18—C19	−0.8 (4)	C35—C36—N3—Ir1	−167.53 (18)
F3—C18—C19—C20	179.3 (3)	C33—C32—N3—C36	−3.2 (3)
C17—C18—C19—C20	−0.1 (5)	C31—C32—N3—C36	178.6 (2)
C18—C19—C20—F4	−177.8 (3)	C33—C32—N3—Ir1	167.98 (18)
C18—C19—C20—C21	1.9 (5)	C31—C32—N3—Ir1	−10.1 (2)
F4—C20—C21—C16	177.0 (3)		
